# Redox Imbalance and Mitochondrial Abnormalities in Kidney Disease—Volume II

**DOI:** 10.3390/biom14080973

**Published:** 2024-08-09

**Authors:** Tram N. Diep, Haoxin Liu, Ying Wang, Yucheng Wang, David Hoogewijs, Liang-Jun Yan

**Affiliations:** 1 Department of Pharmaceutical Sciences, UNT System College of Pharmacy, University of North Texas Health Science Center, Fort Worth, TX 76107, USA; tramdiep@my.unthsc.edu (T.N.D.); haoxinliu@my.unthsc.edu (H.L.); 2 Institute of Medicinal Biotechnology, Chinese Academy of Medical Science and Peking Union Medical College, Beijing 100050, China; wangying@imb.pumc.edu.cn (Y.W.); wangyucheng@imb.pumc.edu.cn (Y.W.); 3 Department of Endocrinology, Metabolism and Cardiovascular System, University of Fribourg, 1700 Fribourg, Switzerland; david.hoogewijs@unifr.ch

The kidney performs fundamental functions by eliminating metabolic waste and reabsorbing essential nutrients and electrolytes such as glucose, proteins, ions, and anions [1,2]. It also participates in the regulation of renal hemodynamics by secreting hormones such as renin, erythropoietin, and the active form of vitamin D [2,3,4]. Moreover, the kidney can carry out miscellaneous functions such as gluconeogenesis under fasting conditions and the catabolism of small peptide hormones [1,2,4]. As such, the kidney is a highly energy-demanding organ that constantly requires active mitochondrial bioenergetics metabolism [5,6]. Any disruption along this cascade of energy generation could cause kidney dysfunction. The kidney features a unique gradient of O_2_, aiming to maintain the tissue partial O_2_ pressure relatively low, but kidney disease leads to an increase in PO_2_ as a consequence of decreased O_2_ consumption. Similarly, a redox imbalance or oxidative stress and mitochondrial abnormalities provoke kidney dysfunction [7]. To further understand the underlying mechanisms of a redox imbalance and mitochondrial abnormalities in kidney disease, we have edited a second volume of the Special Issue of the journal *Biomolecules* titled “Redox Imbalance and Mitochondrial Abnormalities in Kidney Disease— Volume II”. This Special Issue now concludes with a collection of eight papers, among which four are original papers and four are review papers. 

In the paper concerning podocyte injury under hypoxic conditions, Ren et al. [8] demonstrated that arginase-2 (arg-2), a mitochondrial protein [9], promotes hypoxia-induced podocyte injury, whereby arg-2-knockout mice significantly exhibited nephroprotection against hypoxia-induced cell death. Nonetheless, the authors also noted that arg-2 ablation alone was insufficient to protect mice against hypoxia-induced elevation in albuminuria, suggesting that other players are involved in nephroprotection in the absence of arg-2. However, the potential aging-associated arg-2-dependent effect on albuminuria requires further investigation.

With respect to redox metabolism and vascular calcification (VC) in chronic kidney disease (CKD), Carrillo-Lopez et al. [10] found that advanced aortic VC has a lower content of glutathione peroxidase 3. Moreover, in vascular smooth muscles cells (VSMCs), catalase showed an increase in total levels of nuclear-runt-related transcription factor 2 (RUNX2). Nonetheless, catalase in VSMCs also showed a lower percentage of RUNX2 nuclear staining in response to culture media that facilitates calcifying, where mitochondrial redox dysregulation was not observed. The authors did observe an increase in cytoplasm redox state, demonstrating a regulation of RUNX2 subcellular location by catalase in retarding the onset of VC.

On vitamin C (Vc) and its combination use with metals such as Fe^2+^ or Cu^2+^, Jiang et al. [11] demonstrated that the administration of Vc and copper together to mice could trigger systemic oxidative stress and kidney injury. The deleterious effect of the combined use of VC and copper could be mitigated by N-acetyl cysteine and catalase, implicating the involvement of H_2_O_2_ that was produced by a so-called Fenton reaction [12] in this experimental model of kidney injury. Therefore, Vc and copper should not be used together.

On the topic of diabetic nephropathy (DN) that could be aggravated by numerous risk factors such as ischemia [13] and heavy metal toxicity [14], Wai Linn et al. [15] now expanded the list of risk factors with crocodile oil. The authors found that the long-term supplementation of crocodile oil to diabetic rats exacerbated diabetes-induced mitochondrial dysfunction and kidney injury. In particular, the overexpression of those proteins involved in mitochondrial homeostasis was also disturbed. Interestingly, crocodile oil had no deleterious effects on the kidneys of healthy non-diabetic rats, indicating that pre-existing diabetes is a basal condition for crocodile oil’s detrimental effects on the kidney.

In one of the four review articles published in this Special Issue, Yan reviewed the utility of diabetes induced by a combination of two chemicals: nicotinamide and streptozotocin [16]. The author indicated that this model of type 2 diabetes, and hence a model of diabetic kidney disease (DKD), is less time-consuming and less expensive. Moreover, this model of DKD can be used to explore the mechanisms of DKD and can also serve as a platform for testing the protective effects of numerous natural products and antioxidants that could counteract oxidative stress and fibrosis in kidney injury in DKD. In this respect, Patera et al. [17] further overviewed the role of oxidative stress and renal fibrosis in kidney injury. The authors focused on three pathways that link oxidative stress and renal fibrosis, namely hyperglycemia and mitochondrial energy perturbation, the mineralocorticoid signaling pathway, and the hypoxia-inducible factor signaling pathway. Interestingly, hypoxia represents one of the principal consequences of renal fibrosis, since the expansion of fibrotic tissue is associated with the loss of peritubular capillaries, and reduced blood flow, as well as renal anemia. All three pathways have been touted as plausible targets for combating kidney fibrosis and oxidative stress in kidney disease, and for the identification of novel therapeutics.

Moreover, regarding anti-fibrosis and anti-oxidative stress, Tanase et al. [18] reviewed the role of the Nrf2 signaling pathway in DKD. The authors indicated that while numerous natural compounds can activate the Nrf2 signaling pathway in DKD, larger clinical trials in humans on these compounds are lacking, impeding the implementation of these Nrf2-activating compounds for potential clinical applications in fighting DKD. Finally, on strategies used to treat polycystic kidney disease (PKD), Nakashima et al. [19] discussed the role of mitophagy and redox balance in this battle. They suggested that the fine modulation of mitophagy and its signaling cascade, such as peroxisome proliferator activated receptor-α by the use of corresponding agonists, could be a favorable tactic for combating PKD. The authors also highlighted that the manipulation of the gut microbiota conditions may also be harnessed to fight PKD. 

In conclusion, a redox imbalance or oxidative stress and mitochondrial abnormalities are known to be implicated in kidney disease [7,20]. The articles collected in this Special Issue not only further delineate the underlying mechanisms of a redox imbalance and mitochondrial abnormalities in a variety of kidney injuries, but also provide novel insights into the strategies that can be designed to prevent or treat kidney disease.
